# Identification of main-effect quantitative trait loci (QTLs) for low-temperature stress tolerance germination- and early seedling vigor-related traits in rice (*Oryza sativa* L.)

**DOI:** 10.1007/s11032-019-1090-4

**Published:** 2020-01-04

**Authors:** S. Najeeb, J. Ali, A. Mahender, Y.L. Pang, J. Zilhas, V. Murugaiyan, Lakshminarayana R. Vemireddy, Z. Li

**Affiliations:** 1 Rice Breeding Platform, International Rice Research Institute (IRRI), 4031 Los Baños, Laguna Philippines; 2 Mountain Research Centre for Field Crops, Sher-e-Kashmir University of Agricultural Science & Technology (SKAUST), Khudwani, Kashmir 190025 India; 3 State Key Laboratory of Crop Biology, College of Agronomy, Shandong Agricultural University, Taian, 271018 People’s Republic of China; 4 Plant Nutrition, Institute of Crop Sciences and Resource Conservation (INRES), University of Bonn, 53012 Bonn, Germany; 5 Department of Genetics and Plant Breeding, Sri Venkateswara Agricultural College, Acharya NG Ranga Agricultural University, Tirupati, Andhra Pradesh 517502 India; 6 National Key Facility for Crop Gene Resources and Genetic Improvement, Institute of Crop Science, Chinese Academy of Agricultural Sciences (CAAS), Beijing, 100081 People’s Republic of China

**Keywords:** Low-temperature stress, Germination, Early seedling growth stage, Stress index, SNP markers, Quantitative trait loci

## Abstract

An attempt was made in the current study to identify the main-effect and co-localized quantitative trait loci (QTLs) for germination and early seedling growth traits under low-temperature stress (LTS) conditions in rice. The plant material used in this study was an early backcross population of 230 introgression lines (ILs) in BC_I_F_7_ generation derived from the Weed Tolerant Rice-1 (WTR-1) (as the recipient) and Haoannong (HNG) (as the donor). Genetic analyses of LTS tolerance revealed a total of 27 main-effect quantitative trait loci (M-QTLs) mapped on 12 chromosomes. These QTLs explained more than 10% of phenotypic variance (PV), and average PV of 12.71% while employing 704 high-quality SNP markers. Of these 27 QTLs distributed on 12 chromosomes, 11 were associated with low-temperature germination (LTG), nine with low-temperature germination stress index (LTGS), five with root length stress index (RLSI), and two with biomass stress index (BMSI) QTLs, shoot length stress index (SLSI) and root length stress index (RLSI), seven with seed vigor index (SVI), and single QTL with root length (RL). Among them, five significant major QTLs (*qLTG(I)*_*1*_, *qLTGS(I)*_*1–2*_, *qLTG(I)*_*5*_, *qLTGS(I)*_*5*_, and *qLTG(I)*_*7*_) mapped on chromosomes 1, 5, and 7 were associated with LTG and LTGS traits and the PV explained ranged from 16 to 23.3%. The genomic regions of these QTLs were co-localized with two to six QTLs. Most of the QTLs were growth stage-specific and found to harbor QTLs governing multiple traits. Eight chromosomes had more than four QTLs and were clustered together and designated as promising LTS tolerance QTLs (*qLTTs*), as *qLTT*_*1*_, *qLTT*_*2*_, *qLTT*_*3*_, *qLTT*_*5*_, *qLTT*_*6*_, *qLTT*_*8*_, *qLTT*_*9*_, and *qLTT*_*11*_. A total of 16 putative candidate genes were identified in the major M-QTLs and co-localized QTL regions distributed on different chromosomes. Overall, these significant genomic regions of M-QTLs are responsible for multiple traits and this suggested that these could serve as the best predictors of LTS tolerance at germination and early seedling growth stages. Furthermore, it is necessary to fine-map these regions and to find functional markers for marker-assisted selection in rice breeding programs for cold tolerance.

**Electronic supplementary material:**

The online version of this article (10.1007/s11032-019-1090-4) contains supplementary material, which is available to authorized users.

## Introduction

Rice (*Oryza sativa* L.) is one of the primary food crops for more than 3.5 billion people, about half of the global population, and it provides 19% of the dietary energy and 20% of the dietary protein in developing countries (http://ricepedia.org). A total of 90% of the production and consumption of global rice is contributed by Asian countries, with India and China alone accounting for 55% of rice production (Milovanovic and Smutka 2017). However, rice production has a significant impact on global climatic variations through different factors such as water, soil nutrients, temperature, air pollution, and loss of biodiversity, which threaten global food security and sustainability. The Food and Agriculture Organization of the United Nations (FAO) estimates that global food production must be increased by 70% by 2050 to meet the food requirements for the estimated global population of ~ 9.7 billion in 2050 (www.fao.org). In addition to that, several major biotic and abiotic stress factors significantly influence crop yield. Importantly, abiotic stresses such as drought, submergence, high temperature, cold/low temperature, and salinity directly or indirectly affect the physiological status of rice and negatively alter its overall genetic mechanisms and metabolism, often with negative impacts on yield (Wani et al. 2016; Meena et al. 2017; Abhinandan et al. 2018; Sahebi et al. 2018). More than 50% of plant productivity often becomes decreased by various abiotic stresses, which have effects individually or in combination, leading to an alteration in plant growth metabolism and development (Rejeb et al. 2014; Singhal et al. 2016). Compared with other cereal crops, rice is more sensitive to low-temperature stress (LTS)/cold stress (CS) as it has originated from tropical regions (Saito et al. 2001; Hasanuzzaman et al. 2009; Zeng et al. 2017). In the temperate, tropical, and even subtropical rice-growing regions, cold stress adversely affects the rice crop throughout various growth stages, from germination to harvesting, and causes significant yield losses because of poor germination and seedling establishment, stunted growth pattern, non-vigorous plants, vast spikelet sterility, delay in flowering, and lower grain filling (Ranawake et al. 2014; Martínez-Eixarch and Ellis 2015; Schläppi et al. 2017; Shakiba et al. 2017; Liang et al. 2018; Xiao et al. 2018; Najeeb et al. 2019 (unpublished). Therefore, to minimize these yield losses, particularly in cold-affected regions, it is imperative to identify and develop high-yielding rice cultivars showing tolerance of LTS.

The optimum temperature requirement for rice cultivation ranges between 25 and 35 °C (Nagai and Makino 2009; Luo 2011). Uniform fast seed germination and early seedling vigor are important traits for seedling establishment. However, most rice varieties are severely affected during early seedling growth when the temperature falls below 17 °C (Andaya and Mackill 2003a; Lou et al. 2007; Ranawake et al. 2014; Singh et al. 2016). For better and more stable stand establishment, especially under direct-seeding conditions in temperate and sub-temperate regions, cold tolerance at germination is a pre-requisite (Teng et al. 2001; Cruz and Milach 2004; Wang et al. 2016a). Numerous reports have mentioned growth stage-specific criteria to evaluate and select LTS-tolerant rice cultivars (reviewed by Najeeb et al. (2019) unpublished). With the unpredictable changes in the climatic scenario, LTS causes an average yield reduction of 5–10% and sporadically as much as 20–40% in temperate and high-altitude environments largely in Asia, Australia, Africa, Europe, and South and North America (Sthapit and Witcombe 1998; Anbumozhi et al. 2012; Zeng et al. 2017). Worldwide, more than 15 million ha of rice suffer from cold damage at one or more growth stages (Zhang et al. 2014a). Phenotypic evaluation of rice cultivars typically has been studied during the seedling and booting stages that are critical to rice production, particularly in the temperate and high-latitude or high-altitude regions. However, LTS can severely affect the germination rate and early seedling growth (Zhang et al. 2014a). Therefore, phenotypic evaluation of LTS tolerance at the germination stage is especially significant for these regions.

As compared with *indica*, temperate *japonica* rice cultivars are LTS tolerant, but the tolerance mechanism is manifested only at specific growth stages (Glaszmann et al. 1990; Saito et al. 2001; Wang et al. 2016b; Pradhan and Rani 2017). Several rice cultivars with a high degree of LTS tolerance have been bred and released in China, India, the USA, Australia, Japan, Vietnam, Iran, Korea, and Egypt (Ye et al. 2009; da Cruz et al. 2013; Bonnecarrere et al. 2015; Donoso et al. 2015). However, this significant achievement in LTS tolerance has been made only in the *japonica* subspecies. Therefore, developing *indica* cold-tolerant rice cultivars has remained an vital breeding objective, particularly in temperate and sub-temperate target environments (da Cruz et al., 2013; Zhang et al. 2014a; Sales et al. 2017), and it is still a big challenge to plant breeders and biotechnologists.

Conventional plant breeding has achieved partial success in developing LTS-tolerant crops because of the complexity of stress tolerance traits, the low genetic variance of yield-attributing components under stress conditions, and the lack of efficient selection criteria (Sanghera et al. 2011; Singh et al. 2016). Therefore, it is imperative to find complementary strategies to conventional breeding methodologies such as molecular marker technologies, linkage maps, and advanced genomic tools to develop LTS-tolerant rice cultivars. LTS tolerance in rice is a very complex and polygenic trait that is genetically controlled by multiple QTLs. Several LTS tolerance QTLs have been mapped on different genomic regions distributed on all 12 rice chromosomes using various types of molecular markers such as RFLP, AFLP, SSR, STS, and SNP, which has facilitated the identification of chromosomal regions associated with tolerance of low temperature (Andaya and Tai 2006; Lou et al. 2007; Ji et al. 2010; Ye et al. 2010; Jena et al. 2012; Verma et al. 2014; Satoh et al. 2016; Wang et al. 2016a; Jiang et al. 2017).

To date, more than 550 QTLs have been reported for different growth stages (germination, seedling, and reproductive/booting stage) for tolerance of LTS by using DNA-based molecular markers on different genetic backgrounds derived from bi-parental mapping populations and diverse genetic resources of rice accessions (Liang et al. 2018; Najeeb et al. (2019) unpublished). Mapping of the stable QTLs for LTS at the reproductive/booting stage is more of a major challenge than at the seedling stage because of difficulties in accurate phenotypic screening and also the complexity of molecular genetics and physiological pathways. Liang et al. (2018) noted more than 100 QTLs responsible for cold tolerance, particularly in the booting stage. However, despite further progress in the molecular genetic dissection of LTS tolerance in rice, few of them were confirmed to be stable QTLs across environments (Fujino et al. 2008; Ji et al. 2010; Li et al. 2013; Cui et al. 2013; Kim et al. 2014; Zhu et al. 2015). With the development of advanced genomics and molecular marker technology, six QTLs (*qCTS-9*, *qCT8*, *qCTB7*, *qCTB3*, *qCT-3-2*, and *qCTB10-2)* have been fine-mapped via a map-based cloning approach and only two QTLs (*Ctb1* and *CTB4a*) have been cloned and functionally conferred to LTS (Kuroki et al. 2007; Saito et al. 2010; Zhou et al. 2010; Shirasawa et al. 2012; Zhu et al. 2015; Li et al. 2018; Sun et al. 2018). However, even with the different genetic backgrounds of rice germplasm, QTLs for stage-specific cold tolerance were mapped on different locations on the 12 chromosomes. Several researchers have been using different temperature regimes ranging from 4 to 28 °C for cold tolerance screening while employing diverse rice germplasm and bi-parental mapping populations, and they have reported several vegetative- and reproductive-stage QTLs for cold tolerance (Fujino and Matsuda 2010; Suh et al. 2010; Jiang et al. 2011; Park et al. 2013; Zhu et al. 2015; Pan et al. 2015; Shakiba et al. 2017; Zhao et al. 2017; Liang et al. 2018; Sun et al. 2018; Xiao et al. 2018). Through genome-wide association studies (GWAS) approaches, 97 QTLs were identified for LTS tolerance at the germination stage (Pan et al. 2015; Sales et al. 2017; Schläppi et al. 2017). In a similar way, 211 QTLs for seedling-stage tolerance and 72 QTLs for booting-stage tolerance were identified while using diverse rice genetic resources (Pan et al. 2015; Lv et al. 2016; Wang et al. 2016a; Shakiba et al. 2017; Xiao et al. 2018; Zhang et al. 2018b).

Despite the progress in LTS tolerance in rice, few QTLs mapped or cloned have been used in breeding programs due to the possible effects of epistasis and QTL × environment interactions (Li 2001). The major drawback of a breeding program for the development of LTS tolerance is expressed by the inconsistency across varying environments, the huge technical gap between screening in growth chambers and field experiments for quantitatively inherited traits, and the lack of suitable donors (Nagano 1998; Zhang et al. 2014b). Low-temperature germination ability plays a vital role in the success of rice cultivation in temperate and sub-temperate regions. Despite the existence of a considerable amount of genetic variation for germination under LTS, most breeding programs were not able to make any significant breakthrough, possibly because the trait is genetically complex.

To overcome problems in the breeding and molecular dissection of LTS, we have been using an early backcross breeding strategy as green super rice breeding technology (GSR-BT) with selective introgression lines, which we have been using for dissecting for many complex traits and this has been successful in many applications (Ali et al. 2016, 2018; Li et al. 2016; Dimaano et al. 2017; Pang et al. 2017; Feng et al. 2018; Liang et al. 2018). Here, we mainly focused on two targets: (i) development of trait-specific introgression lines (ILs) via an early backcross breeding (BC) approach, and (ii) dissection of the molecular genetics of LTS tolerance traits using the ILs and 6 K genotyping. In the present study, we used a total of 230 BC_1_F_7_ ILs derived from the Weed Tolerant Rice1 (WTR-1) as a recipient parent and Haoannong (HNG) as a donor parent, which were evaluated for LTS tolerance at germination and post-germination stages. Here, we discuss the QTLs and markers related to LTS tolerance at germination and early growth stages in rice that can be used to facilitate marker-assisted breeding through recurrent selection.

## Materials and methods

### Plant materials

The experiment was conducted at the International Rice Research Institute (IRRI), Los Baños, Philippines (14° 11 N, 121° 15 E) during 2015 using an early backcross mapping population of 230 BC_1_F_7_ lines developed by single backcross with recipient parent WTR-1 and HNG as the pollen (donor) parent. The population was developed by using single seed descent method at IRRI, and further detailed information of this breeding scheme and population development were explained in Jewel et al. (2019) and Murugaiyan et al. (2019). Both parents showed 100% germination rate under control conditions (27 °C and 19 °C day/night temperature), whereas, at LT (14 °C/12 °C), the former showed a low germination rate and the latter a high germination rate.

### Phenotypic evaluation of early germination and seedling vigor traits under LTS

One hundred seeds of each line were placed on a double paper towel in 9-cm Petri dishes moistened with double distilled water in a complete randomized design (CRD) with two replications each. To eliminate the effect of dormancy on seed germination, the seed samples were incubated at 50 °C for 1 week before conducting the experiment. The materials were put inside the indoor growth chamber at IRRI adjusted to 14 °C and 12 °C day and night temperature, respectively, with relative humidity fixed at 70%. The light intensity was adjusted at 450 micro mol quanta m^−2^ s^−5^ (normal day-night cycle) was used. Six 1000 W incandescent bulbs were placed inside the chamber at approximately 50 cm above the chamber floor and were adjusted to provide a 13-h light period and 11-h dark period. The moisture level in the Petri plates was routinely checked every day after placing the seed inside the controlled chamber. The control treatment was laid out in the same manner inside the Phytotron facilities provided at IRRI, except for the temperature conditions, which were fixed at the normal range, 27 °C and 19 °C for day and night temperature, respectively. Data were recorded on five traits: low-temperature germination percentage (LTG) (%), shoot length (SL) (mm), root length (RL) (mm), biomass weight (BW) (g), and seedling vigor index (SVI). All traits except BW were recorded in three stages, LTG at 2, 4, and 6 days after sowing (DAS), whereas SL, RL, and SVI were noted at 10, 13, and 16 days after incubation. However, BW was recorded in the last stage only, which was on day 16 of incubation (Table 1).

**Table 1:** Traits’ observations related to seed germination and early seedling vigor traits in rice

S. no.	Trait designation	Stages	Trait description
1	LTG	LTG-I, LTG-II, and LTG-III	The numbers of seeds germinated after 2, 4, and 6 days of sowing were calculated as germination percentage (GP) at low temperature (day/night temperature is 14 °C/12 °C).
2	SL	SL-I, SL-II, and SL-III	Measured from the collar region to the tip of the topmost leaf at stages of 10, 13, and 16 days after incubation at low temperature
3	RL	RL-I, RL-II, and RL-III	Measured from the collar region down to the tip of the longest root at stages of 10, 13, and 16 days after incubation at low temperature.
4	SVI	SVI-I, SV-II, and SVI-III	Seedling vigor index were calculated in 10, 12, and 16 days of sowing by using with formula as SVI = [GP × shoot length (mm)]/100
5	BW	BW-III	Fresh biomass recorded in only last stage which was on the 16th day of incubation
4	LTGS	LTGS-I, LTGS-II, and LTGS-III,	Stress index (SI) of LTG trait were calculated using the following formula as SI = (OSC-ONC)/ONC where OSC = phenotypic observation under stress conditions; ONC = phenotypic observation under normal conditions.
5	RLSI	RLSI-I, RLSI-II, and RLSI-III	Stress index (SI) of RL were calculated using the following formula as SI = (OSC-ONC)/ONC.
6	SLSI	SLSI-I, SLSI-II, and SLSI-III	Stress index (SI) of SL were calculated using the following formula as SI = [(OSC-ONC)/ONC].
7	AGP	AGP-I, AGP-II, and AGP-III	Adjusted germination percent (AGP) = (germination percent under stress condition divided by germination under normal condition) × 100
8	BMSI	BMSI-III	Calculated biomass stress index at 16th days after sowing
9	RGP, SGI, and RGI	RGP (I-II), RGP (II-III), SGI (I-II), SGI (II-III), RGI (I-II), and RGI (II-III)	The relative germination percentage (RGP), shoot growth index (SGI), and root growth index (RGI) were calculated based on the increased growth from stage I to II, and stage II to III used in the data observations.

Germination was defined as the emergence of a radical or plumule of ≥ 1 mm as proposed by Counce et al. (2000). Germination percentage (GP) at LT was calculated as (LTG) = (GN/NG) × 100, where GN = number of germinated seeds and NG = total number of grains (Wang et al. 2010). In a similar way, stress tolerance index (SI) for low temperature germination (LTGS), shoot length (SLSI), root length (RLSI), and biomass (BMSI) was calculated according to the formula SI = (OSC − ONC) / ONC, where OSC is the phenotypic observation under stress conditions and ONC is the phenotypic observation under normal conditions (Cruz and Milach 2004; Bosetti et al. 2012; Zhang et al. 2014a). Values toward zero indicate a lower stress index of a genotype, which means tolerance of LTS, whereas − 1 value indicates no tolerance at all. Furthermore, adjusted germination percentage was calculated as (AGP) = (Germination percentage under stress conditions divided by germination under normal conditions) × 100 (Shakiba et al. 2017). The seedling vigor index (SVI) was calculated by using the formula SVI = LTG × SL (mm) / 100 (Abdul-Baki and Anderson 1973). Similarly, we focused on the relative germination percentage (RGP), shoot growth index (SGI), and root growth index (RGI) that were calculated, based on the increasing the germination rate, shoot and root growth from stage I to II, and stage II to III used in the data observations. The phenotypic data of the traits were analyzed using statistical software “Plant Breeding Tools” version 1.4 (http://bbi.irri.org/products) for descriptive statistics, including mean, standard deviation, minimum and maximum values, coefficient of variation (%), skewness, kurtosis, and the difference between individuals within ILs. Further, mean data over replications for all traits were used for QTL IciMapping (Meng et al. 2015).

### Genotyping and filtering of 6 K SNP data

Fresh young leaves of 230 ILs and parents were collected after 16 DAS. A modified CTAB method was used for DNA extraction (Murray and Thompson 1980), which was quantified by a NanoDrop 8000 spectrophotometer (Thermo Scientific, USA). For the SNP array, DNA concentrations were adjusted at approximately 50 ng/μl, and further processing was done according to the manufacturer’s instructions for the Illumina Infinium assay using a 6 K gene chip in the Genotyping Services Laboratory of IRRI. The resulting intensity data were processed by using genotyping module V2011.1 of Genome Studio software (Illumina Inc., San Diego, CA, USA) for SNP calling. The genotypic data were filtered based on the polymorphic markers between the parents, and we further removed the heterozygous and monomorphic alleles in the parents by filtering using Microsoft Excel.

### QTL mapping

The high quality of 704 SNP markers was distributed on all 12 chromosomes. The degree of polymorphism in each chromosome was calculated based on the number of polymorphic SNPs distributed in the chromosome, divided by the total number of SNP markers. The linkage between SNP marker and traits analysis was performed by using IciMapping4.0 (Meng et al. 2015). Mean values of each phenotypic data point were used as input to detect QTLs by the single marker analysis (SMA) method. The logarithm of odds (LOD) threshold value was obtained based on the permutation test (*n* = 1000; *P* = 0.05) of each trait for claiming the significant QTLs with a LOD score of ≥ 3.0. The additive effect with a positive value indicated that the desirable allele came from the recurrent parent (WTR-1), whereas a negative additive value showed that the desirable allele was received from the donor parent (HNG). Auto-filtration of QTLs concerned with different phenotypic traits was done using Microsoft Excel 2007.

## Results

### Germination and seedling growth parameters

**Fig. 1:**
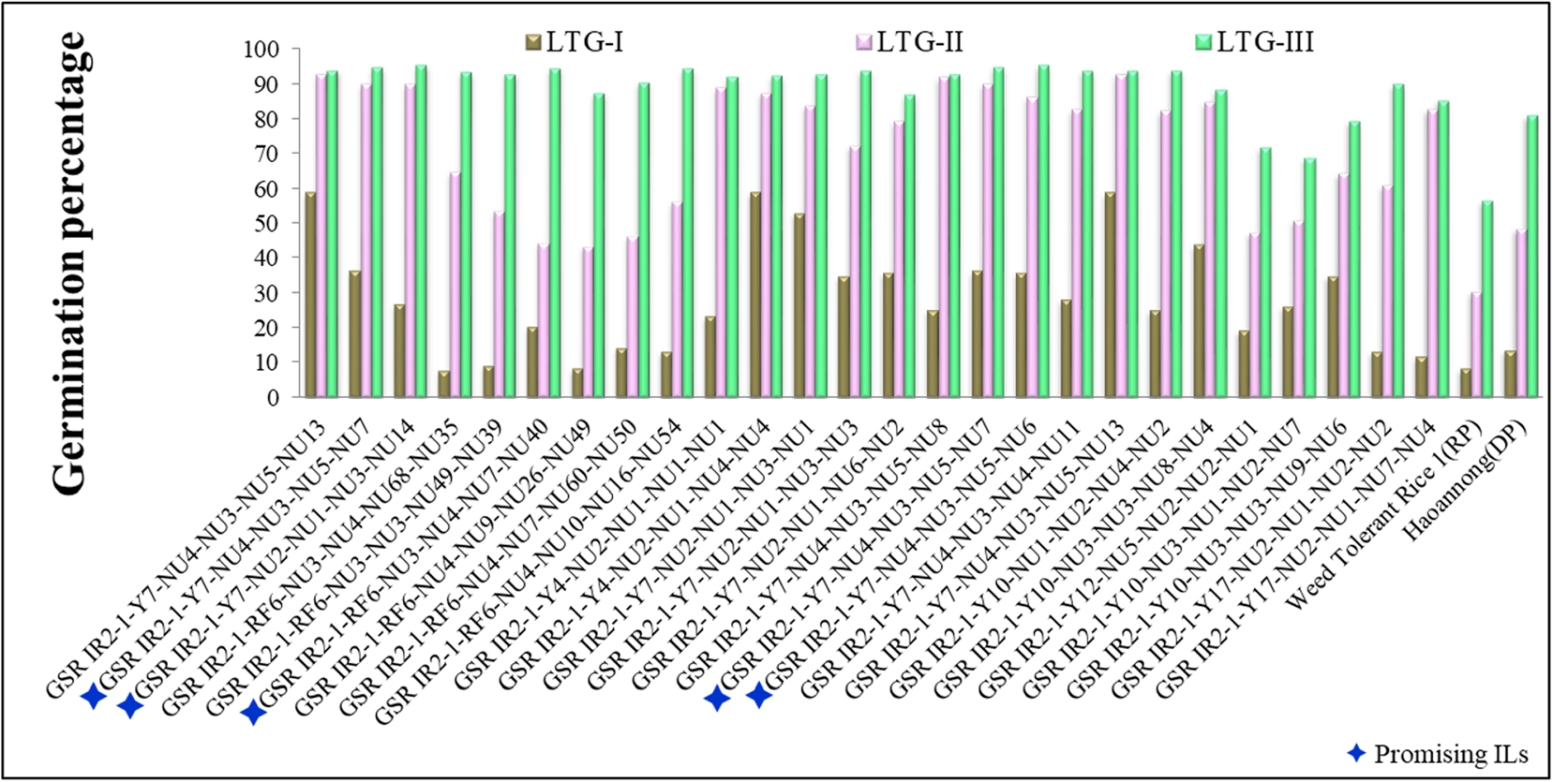
Graphical depiction of 24 promising BILs (~ 12% of the total BIL population) identified for LTS tolerance

**Fig. 2:**
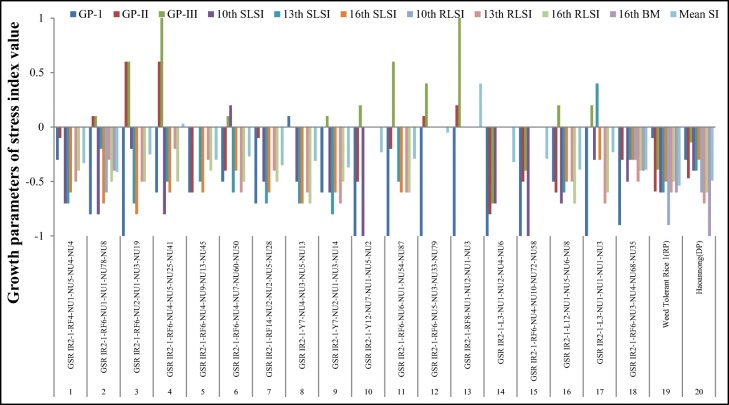
Graphical depiction of 18 promising ILs (~ 7.5% of the total ILs) by low-stress index for all the traits in different growth stages with mean values

The phenotypic variance analysis detected a significant difference in the ILs under LTS and ambient temperature (control) conditions. For the variance between the parents, HNG showed a higher value for LTG, SL, RL, and SVI in all three stages, except at 16 DAS-RL. The results of descriptive statistics of LTG ranges were 0–60%, 0.5–95%, and 0–98% at 2 (LTG-I), 4 (LTG-II), and 6 DAS (LTG-III), whereas − 6.0–89% was recorded for RGP (I-II), 0–60% for RGP (II-III), and 0–101.8% for AGP-I, 0–171.8% for AGP-II, and 0–125.4% for AGP-III in all three stages. Similarly, a wide range in variance was observed for SL (I, 0–20.5 cm; II, 0–33.5 cm; and III, 0–45.6 cm), RL (I, 0–20.9 cm; II, 0–31.5 cm; and III, 0–41.2 cm), and SVI (I, 0–15.6; II, 0–45.8; and III, 0–76.6) at 10, 13, and 16 DAS, respectively (Table 2). The germination rate for all three stages was quite distinct in WTR-1 (8.5%, 43%, and 76%) and HNG (13.5%, 48.5%, and 81.5%) and the high mean germination indicates higher LTS tolerance in the donor parent. As compared to the parents, 19.5%, 34%, and 33% of the ILs recorded higher LTG at 2, 4, and 6 DAS, respectively, whereas some of them were more sensitive to cold stress than WTR-1 due to lower GP, indicating that ILs followed transgressive segregation (Table 2). On the other hand, 24 ILs constituting 10.4% of the total genotypes showed a significantly higher germination rate under cold stress in all the stages (Fig. 1 and Table 3). Of the 24 ILs, five promising lines were identified, *G-1-Y7-NU4-3-5-7*, *G-1-Y7-NU2-1-3-14*, *G-1-Y7-NU4-3-5-6*, *G-1-RF6-NU3-4-7-40*, and *G-I-Y7-NU4-3-5-7*, which showed more than 95% germination under LTS. Moreover, a broad range of variation was observed in ILs for LTG, SL, RL, and SVI, which indicates the scope for the selection of superior genotypes with cold tolerance. Similarly, Fig. 2 represents 7.5% of the ILs (18 lines) showing less than < 0.5 overall mean stress index.

**Table 2:** Comparison of LTS tolerance indicators (phenotypic traits) of the parents and ILs under cold and normal conditions

S. no.	Cold tolerance indicator	Condition	Mean WTR-1	Mean HNG	Mean ± S.D. IL population	Range	Skewness	Kurtosis
1	LTG-I	NC	41.00	97.50	40.80 ± 23.10	3–100	0.37	− 0.68
		SC	8.50	13.80	7.20 ± 11.10	0–60	2.07	4.90
2	LTG-II	NC	89.00	100.00	77.20 ± 20.90	3–100	− 1.77	2.82
		SC	30.60	48.60	44.01 ± 23.50	0–95	0.43	− 0.87
3	LTG-III	NC	89.00	100.00	77.20 ± 20.90	3–100	− 1.77	2.82
		SC	56.70	81.20	60.30 ± 27.80	0–98	− 0.63	− 1.34
4	RL-I	NC	49.10	29.60	41.80 ± 14.80	0–87.6	− 0.25	0.71
		SC	5.40	12.30	6.90 ± 5.80	0–20.9	0.02	− 1.33
5	RL-II	NC	53.90	47.40	58.00 ± 17.20	0–92.2	− 0.99	2.63
		SC	22.00	16.40	12.40 ± 10.00	0–31.5	− 0.19	− 1.49
6	RL-III	NC	54.70	62.30	31.40 ± 35.00	0–95.8	0.34	− 1.65
		SC	28.70	27.10	16.40 ± 13.60	0–41.2	− 0.23	− 1.63
7	SL-I	NC	17.60	20.00	27.60 ± 7.80	0–42.5	− 1.11	1.81
		SC	7.20	11.70	5.60 ± 5.00	0–20.5	0.40	− 0.59
8	SL-II	NC	54.00	42.00	55.10 ± 12.60	0–76.4	− 2.71	9.74
		SC	23.00	24.20	12.70 ± 10.10	0–33.5	− 0.24	− 1.49
9	SL-III	NC	55.20	46.20	30.50 ± 33.10	0–82.3	0.20	− 1.89
		SC	28.20	30.00	17.90 ± 14.80	0–45.6	− 0.23	− 1.66
10	SVI-I	NC	27.30	48.30	33.98 ± 21.20	0–89.7	0.56	− 0.43
		SC	0.80	4.80	1.24 ± 2.50	0–15.6	2.46	8.05
11	SVI-II	NC	96.00	89.40	88.40 ± 30.70	0–150.9	− 1.07	1.31
		SC	33.70	37.40	13.10 ± 12.40	0–45.8	0.48	− 0.92
12	SVI-III	NC	97.70	108.40	49.30 ± 56.00	0–162.3	0.40	− 1.59
		SC	52.80	54.50	25.10 ± 22.80	0–76.6	0.09	− 1.61
13	BW-III	NC	0.50	0.50	0.67 ± 0.30	0–1.53	− 0.28	0.77
		SC	0.20	0.12	0.14 ± 0.12	0–0.6	0.29	− 0.62
14	AGP-I	–	16.85	20.35	13.86 ± 21.09	0–101.80	16.95	16.4
15	AGP-II	–	86.65	91.90	57.53 ± 26.59	0–171.40	3.67	− 1.17
16	AGP-III	–	98.00	95.95	73.74 ± 27.99	0–125.40	− 5.44	− 4.69
17	SGI (I-II)	–	− 0.80	− 0.80	− 0.86 ± 0.21	− 1–0.10	17.43	18.08
18	SGI (II-III)	–	− 0.15	− 0.10	− 0.41 ± 0.31	− 1–0.80	8.18	5.03
19	SGI (I-III)	–	0.00	0.00	− 0.15 ± 0.59	− 1–50	33.11	107.79
20	RGP (I-II)	–	40.50	54.50	36.98 ± 19.69	− 6–89	3.4	− 2
21	RGP (II-III)	–	37.00	18.00	16.27 ± 15.44	0–60	8.77	− 0.25
22	RGI (I-II)	–	10.55	7.00	5.4 ± 5.67	− 3.70–23.90	6.77	− 0.51
23	RGI (II-III)	–	7.75	10.55	3.97 ± 6.16	− 29.80–25.80	− 6.78	23.7

*DAS* days after sowing, *NC* normal (or ambient) conditions, *SC* stress condition. Cold tolerance trait abbreviations were given in Table 1

**Table 3:** Mean performances of 24 ILs identified for high germination rate and low-stress index value under low-temperature stress conditions

S. no.	Type of population	Mean ± SD			Mean stress index
		LTG-I	LTG-II	LTG-III	All traits
1	Promising ILs	29.50 ± 14.90	73.50 ± 16.70	89.80 ± 6.70	− 0.27
2	Entire IL population	7.20 ± 11.10	44.00 ± 23.50	60.30 ± 27.80	− 0.58

Of all the LTS tolerance traits studied in the present experiment in three different stages, LTG-III, 13 DAS-SL, 13 DAS-RL, 16 DAS-SL, and 16 DAS-RL were found to be negatively skewed, while the remaining were observed to be positively skewed under cold stress (Table 2). The kurtosis values were negative and varied from − 0.59 to − 1.66 for 10 DAS-SL and 16 DAS-SL, respectively. Therefore, the shape of the distributions was classified as platykurtic in cold stress as it was less peaky than the Gaussian distribution. All the traits except LTG at stages I and II and SVI followed a normal distribution pattern. The results of ANOVA (analysis of variance) for tolerance traits under LTS conditions are shown in Table 4 and the combination of different growth stages in cold conditions is given in Supplemental Table 1. Among all the traits studied, the largest coefficient of variation (CV) was measured in SGI (II-III) (24.4%), followed by RGI (II-III) (22.3%) and RGII (I-II) (18.7%), and SVI-I (18.0%). A highly significant difference was detected among genotypes and conditions for all the traits. The CV range for five basic traits was observed from 3.21% for RL-III to 24.45% for SGI (II-III). The correlation coefficient was performed initially, using the main traits like LTG, SL, RL, BW, and SVI which were calculated for three stages. Further, the correlation indices of these traits like LTGS, SLSI, RLSI, and BMSI were calculated separately for all three stages. Among these, the correlation coefficients of LTG with BM, RL, SL, and SVI were high and significant (Table 5). Similarly, BM under cold stress demonstrated a strong association with RL, SL, and SVI. Moreover, RL showed a significant and high association with SL (*r = 0.93*), and SVI (*r = 0.88*) in LTG-III and a similar trend was observed between SL and SVI (*r = 0.90*) in LTG-III. On the other hand, non-significant correlations were observed among stress indices, except for the very high and positive association between RLSI and SLSI. The strength of most of the correlations increased markedly toward the later stages. This kind of observation to some extent could be explained by the QTL linkage mapping results, wherein some genomic regions were occupied by QTLs associated with most of these traits under LT conditions.

**Table 4:** Analysis of variance for LTS tolerance traits in three different growth stages in rice

S. no.	Source of variation	DF	Mean squares (variances)				
			LTG	SL	RL	SVI	BW
1	Genotypes	230	3325.1 Pr(< 0.01)	879.2 Pr(< 0.01)	1194.6 Pr(< 0.01)	3140.7 Pr(< 0.01)	0.1
2	Conditions (normal/stress)	1	611,371.4 Pr(< 0.01)	452,520.4 Pr(< 0.01)	698,899.5 Pr(< 0.01)	1,344,434.8 Pr(< 0.01)	32.03 Pr(< 0.01)
3	Stages (I, II, III)	2	462,681.2 Pr(< 0.01)	69,864.1 Pr(< 0.01)	37,737.4 Pr(< 0.01)	258,367.8 Pr(< 0.01)	–
4	Genotype: condition	230	1131.9 Pr(< 0.01)	742.9 Pr(< 0.01)	1044.9 Pr(< 0.01)	2262.9 Pr(< 0.01)	0.05 Pr(< 0.01)
5	Genotype: stage	460	353.3 Pr(< 0.01)	428.4 Pr(< 0.01)	464.2 Pr(< 0.01)	1260.6 Pr(< 0.01)	–
6	Condition: stage	2	32,215.6 Pr(< 0.01)	53,153.0 Pr(< 0.01)	29,551.5 Pr(< 0.01)	172,965.6 Pr(< 0.01)	–
7	Genotype: condition: stage	460	296.4 Pr(< 0.01)	418.1 Pr(< 0.01)	460.5 Pr(< 0.01)	1227.3 Pr(< 0.01)	–
8	Error	1392	5.4	1.8	1.8	6.3	0.004
9	CV (%)		4.48	18.43	14.93	7.14	16.39

**Table 5:** Correlation analysis of the different traits used as cold tolerance phenotypes

	LTG	BM	RL	SL	SVI
LTG	1.000	0.19, (0.41*), [0.51**]	n.s., (0.41*), [0.55**]	n.s, (0.44*), [0.58**]	0.79, (0.75**), [0.76**]
BM		1.000	0.80**, (0.80**), [0.81**]	0.78, (0.84**), [0.84**]	0.42*, (0.72**), [0.76**]
RL			1.000	0.88**, (0.91**), [0.93**]	0.53**), (0.81**), [0.88**]
SL				1.000	0.63**, (0.83**), [0.90**]
SVI					1.000
	LTGS	BMSI	RLSI	SLSI	
LTGS	1.000	n.s.	n.s, (n.s), [n.s]	n.s (n.s.), [n.s.]	
BMSI		1.000	n.s.	n.s	
RLSI			1.000	0.83**, (0.87**), [0.97**]	
SLSI				1.000	

Trait measurements and abbreviations are expanded in Table 1 (Values without brackets represent correlation coefficients for stage I; values inside short brackets for stage II; values inside long brackets for stage III)

### QTLs for LTS tolerance traits

**Fig. 3:**
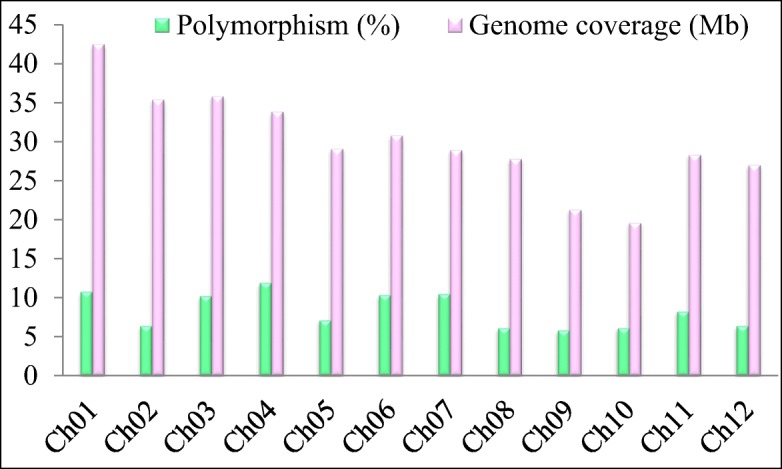
Genomic distribution and genome coverage of polymorphic SNP markers

**Fig. 4:**
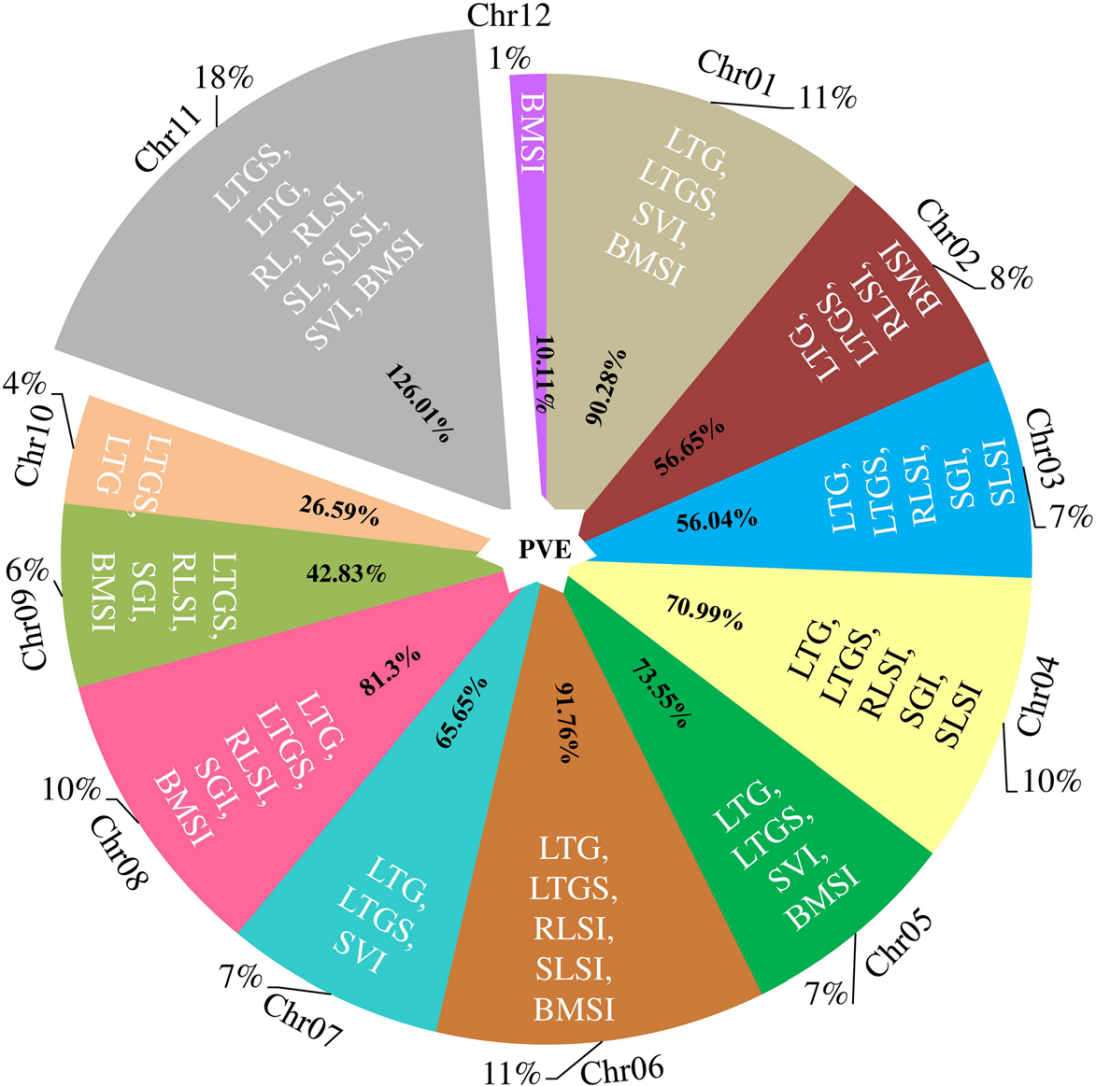
Depicted representation of the distribution pattern of low-temperature stress (LTS) tolerance QTLs associated with phenotypic traits on 12 chromosomes, The total phenotypic variance explained (PVE) of different QTL indicates in the middle part with black color font, and percentage of number QTLs (%) distributed on 12 chromosomes were shown at the outter boundary of pie chart (Chr = chromosome; PVE = phenotypic variation explained; LTG = low-temperature germination; LTGS = low-temperature germination stress index; BMSI = biomass stress index; SL = shoot length; RL = root length; SLSI = shoot length stress index; RLSI = root length stress index; SGI = shoot growth index; SVI = seedling vigor index)

**Fig. 5:**
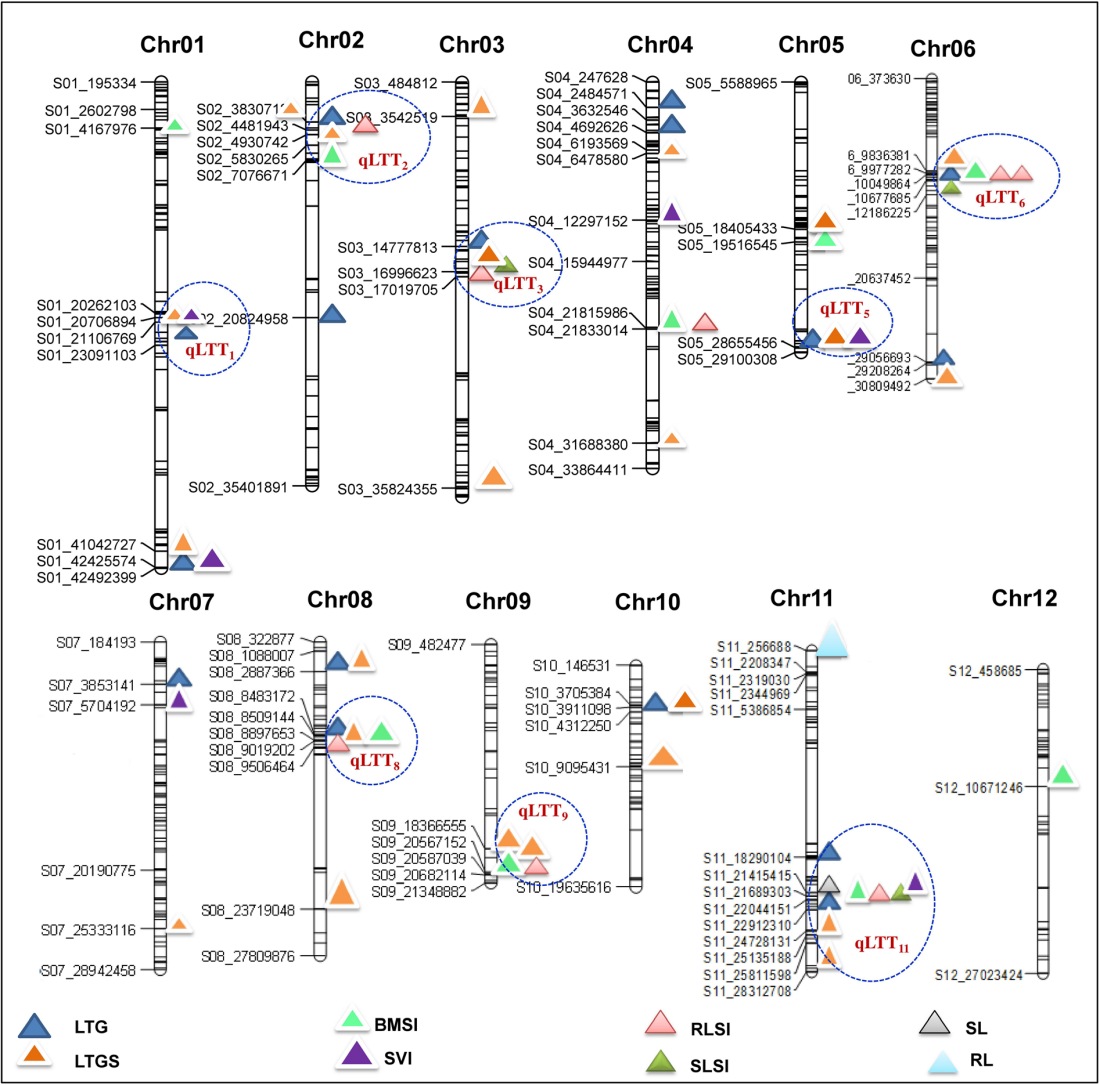
Linkage map and chromosomal position of QTLs (LOD score of ≥ 3) for LTS tolerance in rice. Circles indicate the clustered QTLs located on eight different chromosomes

The linkage map was constructed by using 704 high-quality SNP polymorphic markers, distributed on a total genome size of 353.5 million bases (Mb). The level of polymorphism varied between chromosomes, ranging from 6.1% (chromosome 8) to 11.9% (chromosome 4), and the length of the chromosomes ranged from 19.5 Mb (chromosome 10) to 42.3 Mb (chromosome 1) (Fig. 3). To concentrate on more significant QTLs contributing maximum phenotypic variation (PV) for germination and early seedling growth traits, the critical LOD threshold was set at ≥ 3.0, which is higher than the level used in many other studies, and this also reduced the false positives. A total of 82 QTLs were mapped on the 12 chromosomes associated with 16 LTS tolerance traits in three different growth stages (Table 6). These traits were LTG-I, II, and III; LTGS-I, II, and III; RLSI-I and RLSI-II; BMSI and SVI-I, II, and III; SLSI-I; SL-II; RL-I; and SGI, and they explained PV ranging from 6.95 to 23.38%. The number of QTLs associated with phenotypic traits ranged from one to nine, and these are depicted in Figs. 4 and 5 and listed in Table 6. An average of 6.8 QTLs are distributed on each chromosome, and the highest number of QTLs was found to be located on chromosome 11, containing 15 QTLs, and chromosomes 6 and 1 each had nine QTLs, together explaining average PV of 8.40%, 10.19%, and 10.03%, respectively (Fig. 4). In total, about 67% of the desirable alleles for QTLs were received from the donor parent (HNG), whereas the recipient parent contributed 33% of the favorable alleles. The contribution of M-QTLs regarding PV explained ranged from 10 to 23.38%, and five significant M-QTLs were considered to be major QTLs since the PV explained surpassed 15%. The highest PV explained of 16.57% (*qLTGS(I-II)*_*1*_), 16.08% (*qLTG(I)*_*1*_), 23.38% (*qLTGS(I)*_*5*_), 18.56% (*qLTG(I)*_*5*_), and 20.12% (*qLTG(I)*_*7*_) was located at 41.04 Mb and 42.4 Mb on chromosome 1, at 28.6 Mb on chromosome 5, and at 3.85 Mb on chromosome 7, respectively, and exhibited a mean LOD score of 8.77 (Table 6).

**Table 6:** List of low-temperature stress tolerance M-QTLs and previously mapped QTLs on all 12 chromosomes

S. no.	Trait	QTL	Chr	PVE	LOD	Add	Peak marker	Position (Mb)	Previously reported QTLs and their position	Reference
1	LTG	*qLTG(III)*_*1*_	1	7.88	3.42	8.20	48SNP_1_20706894	20.70		
2	LTGS	*qLTGS(II)*_*1*_	1	7.92	3.44	0.09	48SNP_1_20706894	20.70		
3	LTGS	*qLTGS(III)*_*1*_	1	7.87	3.42	0.18	48SNP_1_20706894	20.70		
4	SVI	*qSVI(I)*_*1*_	1	7.11	3.08	− 1.06	49SNP_1_21106769	21.10	L7-22.4 Mb; ELC2 − 22.6 Mb, *qCTSR-1*-22.9 Mb	(Andaya and Mackill 2003b; Fujino and Matsuda 2010; Lv et al. 2016; Zhang et al. 2018b)
5	SVI	*qSVI(I)*_*1–2*_	1	8.02	3.49	− 1.04	75SNP_1_42425574	42.42	*qCTS1-5*-41.8 Mb	(Ranawake et al. 2014; Shinada et al. 2014)
6	BMSI	*qBMSI*_*1*_	1	7.94	3.45	− 0.12	16SNP_1_4167976	41.16		
7	LTG	*qLTG(I)*_*1*_	1	16.08*	7.31	− 6.58	75SNP_1_42425574	42.42	*Os01g74470*-43.03 Mb	(Shakiba et al. 2017)
8	LTGS	*qLTGS(I)*_*1–2*_	1	16.57^*^	7.55	− 0.12	72SNP_1_41042727	41.04	*qGERM1-8*, *qCTS1-5*, *qSWTPNCT1-4*, and *Os01g71830*-41.5 to 41.7 Mb	(Shakiba et al. 2017)
9	LTGS	*qLTGS(I)*_*1–1*_	1	10.89	4.81	− 0.11	49SNP_1_21106769	21.10		
10	LTG	*qLTG(III)*_*2*_	2	9.63	4.22	13.76	104SNP_2_20824958	20.82		
11	LTGS	*qLTGS(I)*_*2*_	2	7.44	3.22	0.06	86SNP_2_3830712	3.83		
12	LTGS	*qLTGS(III)*_*2*_	2	8.63	3.76	− 0.18	89SNP_2_4930742	4.93		
13	BMSI	*qBMSI*_*2*_	2	9.37	4.10	− 0.10	90SNP_2_5830265	5.83	L21 and *Os02g12540*-6.6 Mb	(Andaya and Mackill 2003a; Shakiba et al. 2017)
14	RLSI	*qRLSI(I)*_*2*_	2	10.00	4.40	− 0.06	88SNP_2_4481943	4.48		
15	LTG	*qLTG(I)*_*2*_	2	11.58	5.13	3.70	86SNP_2_3830712	3.83		
16	LTG	*qLTG(III)*_*3*_	3	12.18	5.41	− 10.03	155SNP_3_14777813	14.77		
17	LTGS	*qLTGS(II)*_*3*_	3	8.88	3.88	0.11	134SNP_3_3542519	3.54	*qCTSR3-1*-3.3 Mb	(Zhang et al. 2018b)
18	LTGS	*qLTGS(III)*_*3*_	3	7.96	3.46	0.20	134SNP_3_3542519	3.54		
19	SGI	*qSGI*_*3*_	3	9.04	3.95	− 2.07	165SNP_3_16996623	16.99		
20	SLSI	*qSLSI(I)*_*3*_	3	7.87	3.42	− 0.07	165SNP_3_16996623	16.99		
21	RLSI	*qRLSI(I)*_*3*_	3	10.11	4.44	− 0.07	166SNP_3_17019705	17.01		
22	LTG	*qLTG(II)*_*4–2*_	4	11.74	5.21	− 8.78	209SNP_4_4692626	4.69		
23	LTG	*qLTG(II)*_*4–1*_	4	9.04	3.95	− 7.67	201SNP_4_2484571	2.48	*qCTS4-1* and *Os04g02410*-1.1 Mb	(Andaya and Mackill 2003b; Shakiba et al. 2017)
24	LTGS	*qLTGS(I)*_*4–1*_	4	9.92	4.36	− 0.07	216SNP_4_6478580	6.47		
25	LTGS	*qLTGS(I)*_*4–2*_	4	8.76	3.82	− 0.12	273SNP_4_31688380	31.68	*qCTS4-2*, L48, *qLTG4* and *Os4g52800*-31.4 Mb	(Andaya and Mackill 2003b; Fujino and Matsuda 2010; Shinada et al. 2014; Lv et al. 2016; Shakiba et al. 2017)
26	RLSI	*qRLSI(I)*_*4*_	4	8.02	3.49	0.06	258SNP_4_21833014	21.83		
27	SGI	*qSGI*_*4*_	4	7.34	3.18	1.74	257SNP_4_21815986	21.81		
28	SVI	*qSVI(II)*_*4*_	4	6.95	3.00	− 3.61	229SNP_4_12297152	12.29		
29	BMSI	*qBMSI*_*4*_	4	9.22	4.03	0.10	258SNP_4_21833014	21.83		
30	LTG	*qLTG(I)*_*5*_	5	18.56	8.56	− 8.23	323SNP_5_28655456	28.65	*qCTB5*, *SWTPNCT5*-27.3 Mb, *qCTSR5-1*-28.03 Mb, *qCTSD5-2*-29.4 Mb	(Andaya and Mackill 2003b; Shakiba et al. 2017; Zhang et al. 2018b)
31	LTG	*qLTG(II)*_*5*_	5	7.27	3.14	− 11.27	323SNP_5_28655456	28.65		
32	LTGS	*qLTGS(I)*_*5*_	5	23.38^*^	11.10	− 0.18	323SNP_5_28655456	28.65		
33	LTGS	*qLTGS(III)*_*5*_	5	8.60	3.75	− 0.18	307SNP_5_18405433	18.40		
34	SVI	*qSVI(I)*_*5*_	5	7.04	3.04	− 1.13	323SNP_5_28655456	28.65		
35	BMSI	*qBMSI-5*	5	8.70	3.79	− 0.10	310SNP_5_19516545	19.51	L51, *qLTG5-2*, *qCTS5*-21.4 Mb	(Andaya and Mackill 2003a; Li et al. 2013; Ranawake et al. 2014; Lv et al. 2016; Jiang et al. 2017)
36	LTG	*qLTG(I)*_*6*_	6	12.16	5.40	− 4.90	365SNP_6_10049864	10.04		
37	LTGS	*qLTGS(II)*_*6*_	6	9.85	4.32	0.11	400SNP_6_30809492	30.80		
38	LTGS	*qLTGS(III)*_*6*_	6	8.89	3.88	0.22	398SNP_6_29208264	29.20	*qCTS6*-23.6 Mb, *qCTSD6-1* and *qCTSR6-1*-24.2 Mb	(Li et al. 2013; Zhang et al. 2018a)
39	RLSI	*qRLSI(II)*_*6*_	6	6.95	3.00	0.06	368SNP_6_10677685	10.67		
40	SLSI	*qCLSI(I)*_*6*_	6	7.46	3.23	0.06	374SNP_6_12186225	12.18		
41	BMSI	*qBMSI*_*6*_	6	9.04	3.95	0.10	364SNP_6_9977282	9.97		
42	LTGS	*qLTGS(I)*_*6*_	6	14.58	6.57	− 0.10	363SNP_6_9836381	9.83		
43	RLSI	*qRLSI(I)*_*6*_	6	10.11	4.44	0.07	364SNP_6_9977282	9.97		
44	LTG	*qLTG(III)*_*6*_	6	12.72	5.67	12.15	398SNP_6_29208264	29.20		
45	LTG	*qLTG(I)*_*7*_	7	20.12^*^	9.37	− 9.37	410SNP_7_3853141	3.85		
46	LTGS	*qLTGS(I)*_*7–1*_	7	14.29	6.43	− 0.15	392SNP_6_20637452	3.94		
47	LTG	*qLTG(II)*_*7–1*_	7	8.25	3.59	− 13.12	410SNP_7_3853141	3.85		
48	LTG	*qLTG(II)*_*7–2*_	7	8.26	3.59	− 10.93	449SNP_7_20190775	20.19	*qCTS7(2)*, *Os07g33480*, *Os07g33600*, *Os07g33670*-19.5 Mb	(Ranawake et al. 2014; Shakiba et al. 2017)
49	LTGS	*qLTGS(I)*_*7–2*_	7	7.32	3.17	− 0.10	464SNP_7_25333116	25.33		
50	SVI	*qSVI(II)*_*7*_	7	7.41	3.21	− 3.69	412SNP_7_5704192	5.70		
51	LTG	*qLTG(III)*_*8*_	8	11.89	5.28	− 9.63	497SNP_8_8509144	8.50	*qCTF8*-8.4 Mb, *qCTGERM8* and *qCTS8-3*-10.5 Mb	(Shinada et al. 2014; Wang et al. 2016a; Shakiba et al. 2017)
52	LTGS	*qLTGS(III)*_*8*_	8	10.23	4.50	− 0.20	500SNP_8_8897653	8.89		
53	BMSI	*qBMSI*_*8*_	8	11.94	5.30	− 0.12	501SNP_8_9019202	9.01		
54	RLSI	*qRLSI(I)*_*8*_	8	7.90	3.43	− 0.06	503SNP_8_9506464	9.50		
55	SGI	*qSGI*_*8*_	8	8.18	3.56	2.02	514SNP_8_23719048	23.71		
56	LTGS	*qLTGS(I)*_*8*_	8	10.38	4.57	0.07	480SNP_8_2887366	2.88		
57	LTGS	*qLTGS(II)*_*8*_	8	10.02	4.40	0.11	514SNP_8_23719048	23.71		
58	LTG	*qLTG(I)*_*8*_	8	10.76	4.75	3.76	480SNP_8_2887366	2.88		
59	LTGS	*qLTGS(III)*_*9*_	9	10.08	4.43	− 0.19	555SNP_9_20587039	20.58		
60	RLSI	*qRLSI(I)*_*9*_	9	10.00	4.39	− 0.06	556SNP_9_20682114	20.68		
61	LTGS	*qLTGS(I)*_*9*_	9	7.51	3.25	0.06	551SNP_9_18366555	18.36		
62	SGI	*qSGI*_*9*_	9	8.06	3.50	− 1.80	555SNP_9_20587039	20.58		
63	BMSI	*qBMSI*_*9*_	9	7.18	3.11	− 0.09	554SNP_9_20567152	20.56		
64	LTGS	*qLTGS(I)*_*10*_	10	7.34	3.18	0.06	567SNP_10_3911098	3.91		
65	LTGS	*qLTGS(III)*_*10*_	10	7.25	3.14	− 0.17	582SNP_10_9095431	9.09	*qLTG-10*-9.81 Mb, *qCTSD10-1*-10.5 Mb and *qCTSR10-1*-11.02 Mb	(Jiang et al. 2017; Zhang et al. 2018b)
66	LTG	*qLTG(I)*_*10*_	10	12.00	5.33	3.78	566SNP_10_3705384	3.70		
67	RLSI	*qRLSI(I)*_*11*_	11	10.91	4.81	− 0.07	642SNP_11_21415415	21.41		
68	LTG	*qLTG(I)*_*11–1*_	11	7.73	3.35	− 4.65	630SNP_11_18290104	18.29	*qCTS11(2)-2* and *Os11g33330*-19.6 Mb	(Ranawake et al. 2014; Shakiba et al. 2017)
69	LTG	*qLTG(I)*_*11–2*_	11	9.55	4.19	3.37	651SNP_11_22912310	22.91		
70	LTG	*qLTG(II)*_*11*_	11	9.74	4.27	− 8.75	607SNP_11_2344969	2.34		
71	LTG	*qLTG(III)*_*11*_	11	8.68	3.78	− 9.55	604SNP_11_2208347	2.20		
72	LTGS	*qLTGS(I)*_*11*_	11	7.57	3.28	0.07	652SNP_11_24728131	24.72		
73	LTGS	*qLTGS(III)*_*11*_	11	8.86	3.87	− 0.18	656SNP_11_25811598	25.81		
74	RL	*qRL(I)*_*1*_	11	7.22	3.12	− 1.69	602SNP_11_256688	0.25		
75	RLSI	*qRLSI(II)*_*11*_	11	7.52	3.26	− 0.06	643SNP_11_21689303	21.68		
76	SL	*qSL(II)*_*11*_	11	7.62	3.30	− 2.93	643SNP_11_21689303	21.68		
77	SGI	*qSGI*_*11*_	11	8.52	3.71	− 1.91	643SNP_11_21689303	21.68		
78	SLSI	*qSLSI(I)*_*11*_	11	7.16	3.10	− 0.06	643SNP_11_21689303	21.68		
79	SVI	*qSVI(II)*_*11*_	11	7.66	3.32	− 3.66	602SNP_11_256688	0.25		
80	SVI	*qSVI(III)*_*11*_	11	8.08	3.51	− 6.73	642SNP_11_21415415	21.41		
81	BMSI	*qBMSI*_*11*_	11	9.19	4.02	− 0.10	646SNP_11_22044151	22.04		
82	BMSI	*qBMSI*_*12*_	12	1011	4.44	− 0.13	685SNP_12_10671246	10.67		

*Chr* chromosome, *PVE* phenotypic variation explained, *LTG* low-temperature germination, *LTGS* low-temperature germination stress index, *BMSI* biomass stress index, *SL* shoot length, *RL* root length, *SLSI* shoot length stress index, *RLSI* root length stress index, *SGI* shoot growth index, *SVI* seedling vigor index, *LOD* Logarithm of odds, *Add* additive effect

*Major QTLs

### QTLs for LTS germination and stress index at three stages

A total of 21 and 26 QTLs associated with LTG and LTGS. These QTLs were located on all the chromosomes, except for chromosomes 12 (Table 6). Out of these QTLs, nine were found to be involved in LTG-I and six QTLs each for LTG-II and -III, whereas in LTGS, 13 QTLs for LTGS-I, four QTLs for LTGS-II, and nine QTLs for LTGS-III, respectively. Among them, the highest PV was explained by five QTLs (*qLTG(I)*_*1*_, *qLTGS(I)*_*1–2*_, *qLTG(I)*_*5*_, *qLTGS(I)*_*5*_, and *qLTG(I)*_*7*_), on chromosomes 1, 5, and 7, respectively and both of these QTLs received desirable alleles from the donor parent (Table 6). Interestingly, the genomic segments at 28.6 Mb and 3.85 Mb on chromosomes 5 and 7 were shared by germination stage II QTLs (*qLTG(II)*_*5*_ and *qLTG(II)*_*7*_), whereas the chromosome segments occupied by *qLTG(II)*_*11*_, a stage II QTL on chromosome 11, were found to overlap with *qLTG(III)*_*11*_, a stage III QTL. However, the majority of the favorable alleles of such QTLs were contributed by HNG.

In total, nine QTLs each for RLSI and BMSI, seven QTLs for SVI, five QTLs for SGI, three QTLs for SLSI, and a single QTL each for RL and SL, and these QTLs explained PV ranging from 6.95 to 23.38% (Figs. 4 and 5). Among them, 22 minor QTLs with PVE ranging from 6.95% (*qRLSI(II)*_*6*_) to 9.37% (*qBMSI(II)*_*2*_), whereas five of them are major-effect QTLs explained the PV more than 10% (Table 6). These major QTLs (*qRLSI(I)*_*2*_, *qRLSI(I)*_*3*_, *qRLSI(I)*_*6*_, *qRLSI(I)*_*9*_, and *qRLSI(I)*_*11*_) were located on specific genomic regions on five different chromosomes, 4.48 Mb (chromosome 2), 17.01 Mb (chromosome 3), 9.97 Mb (chromosome 6), 20.6 Mb (chromosome 9), 21.41 Mb (chromosome 11), influenced RLSI, whereas two other QTLs (*qBMSI(I)*_*8*_ and *qBMSI(I)*_*12*_) at 9.01 Mb on chromosome 8 and 10.67 Mb on chromosome 12 influenced BMSI. Of the 18 QTLs, 72% of the favorable alleles came from the recipient parent and were located on eight chromosomes (1, 2, 3, 5, 8, 9, 11, and 12), whereas 27% of such alleles, observed on chromosomes 4 and 6, were received from the donor parent.

### Co-localization and clustered QTLs

It was remarkable to note that 16 genomic regions were found to harbor two to four co-localized M-QTLs associated with several traits (Fig. 5 and Table 6). Three genomic regions mapped onto chromosomes 1, 5, and 11 were observed to carry QTLs associated with no fewer than three traits. An M-QTL at the 20.70 Mb region on chromosome 1 was found to be co-localized with three QTLs, *qLTGS(II)*_*1*_, *qLTGS(III)*_*1*_, and *qLTG(III)*_*1*_, that explained a PV of 7.92%, 7.87%, and 7.88%. Similarly, the M-QTLs on chromosome 5 at the 28.6 Mb region harbored four QTLs, LTG (I), LTG (II), LTGS (I), SVI (I), together explaining 56.25% of the PV. Another co-mapped M-QTL on chromosome 11 at the 21.68 Mb region was concomitant with four traits, SL, SGI, SLSI, and RLSI, and explained 30.82% of the total PV.

The co-localized QTLs *qLTG*_*(I)*_ and *qSVI*_*(I*)_ at 42.4 Mb on chromosome 1, *qSGI* and *SLSI*_*(I*)_ at 16.9 Mb on chromosome 3, *qLTG*_*(I)*_ and *qLTG*_*(II*)_ at 3.85 Mb on chromosome 7, *qLTGS*_*(III)*_ and *qSGI* at 20.58 Mb on chromosome 9, *qRL* and *qSVI*_*(II)*_ at 0.25 Mb on chromosome 11, and another two QTLs (*qRLSI*_*(I)*_ and *qSVI*_*(III)*_) at 21.41 Mb on chromosome 11 explained an average PV of 10.11% and the favorable alleles were contributed by the donor parent. Similarly, co-localized regions harboring QTLs *qLTG*_*(I)*_ and *qLTGS*_*(I*)_ at 3.83 Mb on chromosome 2, *qLTGS*_*(II)*_ and *qLTGS*_*(III*)_) at 3.54 Mb on chromosome 3, *qRLSI*_*(I)*_ and *qBMSI* at 21.8 Mb on chromosome 4, *qRLSI*_*(I)*_ and *qBMSI* at 9.97 Mb and another two QTLs, *qLTG*_*(III)*_ and *qLTGS*_*(III*)_ located at 29.2 Mb on chromosome 6, *qLTG*_*(I)*_ and *qLTGS*_*(I*)_ at 2.88 Mb and another two QTLs, *qLTGS*_*(II)*_ and *qSGI*, at 23.7 Mb on chromosome 8, received desirable alleles from the recipient parent and explained an average PV of 9.51%. Most of the M-QTLs were growth stage-specific and located on different chromosomes and they also governed multiple traits together. Eight chromosomes (1, 2, 3, 5, 6, 8, 9, and 11) had no fewer than four QTLs clustered together and were designated as promising LTS tolerance QTLs (*qLTTs*), as *qLTT*_*1*_, *qLTT*_*2*_, *qLTT*_*3*_, *qLTT*_*5*_, *qLTT*_*6*_, *qLTT*_*8*_, *qLTT*_*9*_, and *qLTT*_*11*_, and they explained average of PV ranging from 8.33% (chromosome 1) to 14.06% (chromosome 5).

## Discussion

Germination and early seedling growth traits are governed by a combination of multiple QTLs and genes. The complex inheritance and phenotypic expression are significantly affected by environmental factors (Cruz et al. 2006; Satoh et al. 2016; Shakiba et al. 2017; Zhao et al. 2017). Genetic dissection of potential chromosomal regions harboring QTLs related to LTS tolerance during germination and the early seedling stage is expected to reveal the genetic control of the trait. Therefore, the identification of QTLs with main effects and co-localized QTLs associated with genomic regions governing multiple traits will prove highly valuable for marker-assisted breeding (MAB) for the enhancement of LTS tolerance in rice.

Among the various abiotic stress factors, LTS is one of the major limitations of rice production, particularly in temperate and subtropical regions (Van Nguyen and Ferrero 2006; Zeng et al. 2017). Below 17 °C, rice is severely affected, mainly resulting in poor germination and seedling establishment, a severe reduction in growth, and lower yield (Andaya and Mackill 2003b; Koseki et al. 2010). Despite this fact, some rice cultivars, particularly *japonica* types, are known to possess LTS tolerance (Lou et al. 2007). However, some *indica* cultivars are shown to be more LTS tolerant than *japonica* cultivars (Huang et al. 2012; Pan et al. 2015). Recently, Najeeb et al. (2019) reviewed the LTS tolerance QTLs reported in rice at germination and seedling and booting stages while using different genetic backgrounds such as DHs, RIL_S_, BILs, BC_2_F_1_, F_2_, and F_8_ as mapping populations. Among these populations, a few major QTLs have been identified by Andaya and Mackill (2003b), Fujino et al. (2004), Lou et al. (2007), Baruah et al. (2009), Shinada et al. (2014), Jiang et al. (2017), and Shakiba et al. (2017). However, there are no significant main-effect QTLs related to the interaction of different QTLs for germination and seedling growth traits. Detailed information on M-QTLs and co-localized QTLs associated with multiple traits may provide a better understanding of LTS tolerance, and this is an essential step toward developing cultivars with superior LTS tolerance and enhancing rice production in regions where low-temperature limits rice yield.

### Phenotypic and genotypic variation of LTS tolerance traits

In the current study, significant phenotypic variations were observed in the ILs for cold tolerance and a tendency of most of the traits in LTG and SVI stages toward a normal distribution. The ILs exhibited transgressive segregation for all the traits and mean values of the ILs were found to be intermediate between those of the parental lines, WTR-1 and HNG (Table 2), suggesting a polygenic inheritance of the traits. The pattern of transgressive segregation was, in fact, the contribution of either of the parents in the form of favorable or unfavorable alleles for specific traits in the ILs (Rieseberg et al. 2003). A strong positive correlation was observed in LTG with RL, SL, BM, and SVI. Further, among the ILs, 24 promising lines showed a significantly higher germination rate under cold stress in all three stages (Fig. 1). The high variability in LTG, SL, RL, and SVI in the ILs and their normal distribution pattern were considered for selecting better genotypes and for undergoing QTL analysis for cold tolerance. The positive skewness of LTG-I, LTG-II at 2 and 4 DAS, and SL-I, RL-I, and SVI-II at 10 DAS, SVI at 13 DAS, and SVI and BW at 16 DAS suggests complementary gene action while the negative skewness of LTG-III, SL-II, and RL-II at 13 DAS and SL-III and RL-III at 16 DAS is associated with duplicate (additive × additive) gene action. The strong relationship of LTG, RL, BM, RLSI, and SVI indicates the possibility of common QTLs/genes regulated at the molecular level.

Genetic analysis of LTS tolerance revealed a total of 82 QTLs associated with 16 traits in three different growth stages and they were distributed on all 12 chromosomes. The PV explained ranged from 6.95 to 23.38% and LOD value from 3.0 to 11.1, while no QTLs were identified for the remaining 12 traits (SL-I, III; SLSI-II, III; RL-II, III; RLSI-III; AG-I, II, and III; RGR-I and II) perhaps due to the non-significant difference among the ILs for such traits. Of these 82 QTLs, 27 main-effect QTLs (M-QTLs) identified on 12 chromosomes. These QTLs were significantly associated with LTG, LTGS, RLSI, and BMSI. Among the total number of QTLs, only five were identified as major M-QTLs (LOD > 7.0 and PV explained > 15%) and they were governing germination-related traits LTG(I)_1_ (LOD 7.31 and 16.08%), LTGS(I)_1–2_ (LOD 7.55 and 16.57%), LTG(I)_5_ (LOD 8.56 and 18.56%), LTGS(I)_5_ (LOD 11.1 and 23.38%), and LTG(I)_7_ (LOD 9.3% and 20.12%) on three chromosomes (1, 5, and 7), respectively. The favorable alleles for all these major QTLs were contributed by HAN, indicating the possibility of using this variety as a parent in future breeding programs. In different growth stages, the stability of M-QTLs is an imperative factor when deciding on the use of QTLs in breeding programs. The major M-QTLs governing germination rate in two stages, LTG(I) and LTG(II), were identified on chromosome 5 in the genomic region 28.6 Mb. Another major stable M-QTL was identified on chromosome 7 (3.85 Mb) for LTG(I) and LTG(II) with PV explained of 20.12% and 8.25% and LOD value of 9.37 and 3.59. Likewise, a few M-QTLs with relatively higher PV explained were consistently governing two stages, such as LTGS(II) and LTGS(III) (20.70 Mb) on chromosome 1 and LTGS(II) and LTGS(III) on chromosome 3. In earlier reports, Fujino et al. (2008), Li et al. (2013), Cui et al. (2013), Shinada et al. (2014), and Zhu et al. (2015) mentioned a few stable QTLs across several environments under LTS. The importance of stable QTLs is that they have larger additive effects and are less affected by the environment.

Co-localized and pleiotropic M-QTLs located in adjacent regions on the chromosome govern multiple traits, which are most prominent for the concurrent improvement of multiple traits (Shakiba et al. 2017; Li et al. 2018; Liang et al. 2018). In this study, eight clusters of M-QTLs or pleiotropic M-QTLs were identified on different chromosomes governing simultaneously different traits: LTG(III) + LTGS(I) + LTGS(II) + LTGS(III) + SVI(I) (chromosome 1), LTG(I) + LTGS(I) + LTGS(III) + RLS(I) + BMSI (chromosome 2), LTG(III) + RLSI(I) + SGI + SLSI(I) (chromosome 3), LTG(I) + LTG(II) + LTGS(I) + SVI(I) (chromosome 5), LTG(I) + LTGS(I) + RLSI(I) + RLSI(II) + BMSI (chromosome 6), LTG(III) + LTGS(III) + RLSI(I) + BMSI (chromosome 8), LTGS(III) + RLSI(I) + SGI + BMSI (chromosome 9), and RLSI(I) + RLSI(II) + SL(II) + SLSI(I) + SVI(III) + BMSI (chromosome 11). From the total of 61 genomic regions, 16 regions were associated with two to four germination- and seedling growth-related traits, and they were co-localized. These co-localized QTLs were identified in earlier studies on cold tolerance germination and yield component traits in rice (Andaya and Mackill 2003a, b; Koseki et al. 2010; Wang et al. 2016a; Shakiba et al. 2017). Several major low-temperature germination and seedling-stage QTLs have been distributed widely on all 12 chromosomes using bi-parental mapping populations and diverse rice genetic resources (Teng et al. 2001; Chen et al. 2006; Liang et al. 2006, 2018; Fujino et al. 2008; Pan et al. 2015; Jiang et al. 2017; Shakiba et al. 2017; Li et al. 2018). Among the total of 82 M-QTLs, 67% of the M-QTLs received favorable alleles from donor parent HNG, which is significant in LTS tolerance. Therefore, HNG is a novel potential resource for cold tolerance. Similarly, several researchers have used different sources of rice cultivars against cold tolerance such as Dongxiang wild rice (*Oryza rufipogon*) (Mao et al. 2015; Yu et al. 2018), Kasalath (Miura et al. 2001), AAV002863 (Lou et al. 2007), USSR 5 (Li et al. 2013), Italica Livorno (Fujino et al. 2004), M-202 (Andaya and Mackill 2003a), Kunmingxiaobaigu (Zhou et al. 2010), Hokkai-PL9 (Kuroki et al. 2007), Lijiangxintuanheigu (Zhang et al. 2014b), 2014), Hyogo-kitanishiki (Ranawake et al. 2014), and variety *Geng* (Meng et al. 2013).

Previously identified major QTLs *qCTS-2* (PVE 27.42%) (Lou et al. 2007) and *qCTS2(2)* (PVE 22.9%) (Ranawake et al. 2014) were also identified in the present study in the form of clustered QTLs harbored by chromosome 2. Similarly, on chromosome 3 (*qLTG-3-1*, 35.1%, and *qLTG-3-2*, 19.3%) (Fujino et al. 2004), chromosome 4 (*qCTS4-1*, 20.8%) (Andaya and Mackill 2003a), chromosome 6 (*qCTS6-1*, 15.3%) (Andaya and Mackill 2003b), chromosome 7 (*qCTS7(2)*, 35.3%; Ranawake et al. 2014, and *qCTS7-1*, 21%; Zhou et al. 2010), chromosome 8 (*qCTB8*, 26.6%) (Kuroki et al. 2007), chromosome 10 (*qRC10-2*, 32.1%) (Xiao et al. 2014), chromosome 11 (*qCTS-11*, 23.1%; Zhang et al. 2014b; and *qCTS11(1)-1*, 22.2%, and *qCTS11(1)-2*, 35.6%) (Ranawake et al. 2014), and chromosome 12 (*qCTS12a*, 40.6%) (Andaya and Mackill 2003a) were significantly allied in the clustered M-QTLs.

### Prime M-QTLs for future breeding programs

In the present study, 82 QTLs were mapped on 61 genomic regions, with an average PV of 9.65%. Eight chromosomes (1, 2, 3, 5, 6, 8, 9, and 11) harbored more than four LTS tolerance QTLs (*qLTTs*). Also, 76.9% of the favorable alleles with additive effects were contributed by donor parent HNG to the total QTLs. The average PV explained by the nine QTLs (8.45%) on chromosome 11 at 21.41 to 22.04 Mb of the M-QTLs and co-localized with QTLs related to LTG, RLSI, SVI, SLSI, SL, and SGI. The present results are closely associated with the early seedling growth-related QTLs *qCTS11(1)-2* and *qCTS11(2)-2* (Ranawake et al. 2014) and germination QTL *qCTGERM11-5* under cold stress (Shakiba et al. 2017). Further, two major M-QTLs, *qLTG(I)*_*1*_ (16.08%) and *qLTGS(I-II)*_*1*_ (16.57%), on chromosome 1 at the 41.0 to 42.4 Mb region were clearly associated with the germination and seedling tolerance QTLs *qCTGERM1-8* and *qCTS1-5* and the other QTLs *qSW1-1 to qSW1-4* (seed weight per plant) and *qSWTCT1-1* (seed weight per panicle) expressed in the same genomic region under cold stress at the reproductive stage (Cui et al. 2013; Yang et al. 2016; Shakiba et al. 2017). Similarly, two other QTLs, *qLTG(I)*_*5*_ (18.56%) and *qLTGS(I)*_*5*_ (23.38%), on chromosome 5 at the 28.65 Mb region were concomitant with *qSWTNCT5* (seed weight per panicle), *qCTB5* (cold tolerance at booting stage), and seedling-stage QTLs *qCTSR5-1* (cold tolerance seed survival rate) and *qCTSD5-2* (cold tolerance severity of damage) (Andaya and Mackill 2003b; Ranawake et al. 2014; Shakiba et al. 2017; Zhang et al. 2018b). Lastly, *qLTG(I)*_*7*_ (20.12%) located on chromosome 7 at 3.85 Mb is a novel locus, which is co-localized with second germination stage QTL *LTG(II)* (8.25%). To date, there is no previously reported QTL on this genomic position, but Ranawake et al. (2014) observed a major QTL, *qCTS7(2)*, on the same chromosome at the 17.4 Mb position, which is associated with seedling-stage cold stress. Furthermore, the M-QTLs identified in our study are very precise and stable because phenotyping of all traits was carried out in a growth chamber for a prolonged period (16 days) as compared to the relatively shorter periods of cold treatment in earlier studies (Lee 2001; Han et al. 2004; Chen et al. 2006; Bosetti et al. 2012; Pouramir et al., 2013; Xiao et al. 2014; Xie et al. 2014). It is noteworthy that, so far no QTLs have been detected for LTS germination associated with SVI and BMSI in the near genomic regions/co-localization of M-QTLs. On chromosome 1 (20.7 Mb) and chromosome 5, QTLs are co-localized governing traits such as LTG, LTGS, and SVI, and on chromosome 11, they are co-localized with LTG, SVI, BMSI, SLSI, and SGI at the 21.6–22.0 Mb position. Therefore, co-localized/clustered QTLs of genomic regions governing multiple traits are most useful for introgression into elite high-yielding breeding varieties to breed for LTS tolerance, particularly for germination and early seedling growth stages.

### Putative candidate genes of major M-QTLs

Among the five major M-QTLs (> 15% PVE), two QTLs each on chromosome 1 (41.04 Mb), and chromosome 5 (28.65 Mb), and single QTL on chromosome 7 (3.85 Mb) are co-localized with M-QTLs on 16 genomic regions were fine-tuned for their peak marker position to identify possible candidate genes using the Rice Annotation Project Database (RAP-DB, http://rapdb.dna.affrc.go.jp/). There are three putative genes for major M-QTLs and 11 gene for co-localized QTLs of each chromosome (Table 7). The QTLs with largest PV on chromosome 5 at 28.65 Mb (23.38%) had possible candidate gene LOC_Os05g49970 encoding translation initiation factor-2 (*eIF2*) involved in various growth developmental stages such as xylogenesis, flowering, sporogenesis, and germination, and also interacting with various abiotic stresses (Martínez-Silva et al. 2012; Mutuku et al. 2015) and hormonal signaling pathways in plant defense mechanisms (Lv et al. 2014). The *eIF2* involved in the physiological and metabolic pathway is rapidly expressed during the germination process such as water uptake, mobilization, and the breakdown of storage reserves of energy compounds for the expansion of the embryo (Dogra et al. 2015; Qian et al. 2015). Further, it is implicated in the formation of the largest *eIF3*, the 40S subunit, which is responsible for recruiting mRNA to the 43S pre-initiation complex and recognizing AUG during protein synthesis (Hinnebusch 2006; Wang et al. 2016c). A recent study of Wang et al. (2016c) mentioned that the *OseIF3e* gene significantly regulates seedling growth during the vegetative stage and also influences the development of organ size and pollen maturation.

**Table 7:** List of possible putative candidate genes in major and co-localized QTL positions

S. no.	Chr	Gene	PVE (%) in M-QTLs	CDS coordinates (5′-3′)	Near to peak marker	Nucleotide length (bp)	Protein length (aa)	Function
M-QTLs putative genes								
1	1	LOC_Os01g73160	16.08	42,427,258–42,425,424	1 kb	540	180	40S ribosomal protein S10, putative, expressed
2	5	LOC_Os05g49970	23.38	28,654,730–28,658,420	726 bp	2052	684	Translation initiation factor IF-2, chloroplast precursor, putative, expressed
3	7	LOC_Os07g07690	20.12	3,859,755–3,868,364	12 kb	4329	1443	PHD-finger domain-containing protein, expressed
Co-localized M-QTLs putative genes								
1	1	LOC_Os01g37100	23.67	20,707,408–20,705,750	1.1 kb	1326	442	RWP-RK domain-containing protein, putative, expressed
2	2	LOC_Os02g07430	19.02	3,837,135–3,833,129	3.2 kb	783	261	OsMADS29 - MADS-box family gene with MIKCc type-box, expressed
3	3	LOC_Os03g06950	16.84	3,550,714–3,540,151	2.3 kb	4740	1580	Ubiquitin carboxyl-terminal hydrolase domain-containing protein, expressed
4	3	LOC_Os03g29810	16.91	16,994,268–16,997,742	1.1 kb	780	260	OsClp6 - putative Clp protease homolog, expressed
5	4	LOC_Os04g35820	17.24	21,829,938–21,830,249	2.7 kb	312	104	Expressed protein
6	6	LOC_Os06g17220	19.15	9,974,991–9,976,880	402 bp	480	160	UDP-glycosyltransferase, putative, expressed
7	6	LOC_Os06g48300	21.61	29,210,947–29,208,086	178 bp	984	328	Protein phosphatase 2C, putative, expressed
8	8	LOC_Os08g05440	21.14	2,878,953–2,890,634	3.2 kb	4497	1499	NB-ARC domain-containing protein, expressed
9	8	LOC_Os08g37444	18.20	23,722,359–23,717,585	1.4 kb	1875	625	Signal recognition particle receptor, putative, expressed
10	9	LOC_Os09g35780	18.11	20,588,660–20,587,580	541 bp	582	194	BAP2, putative, expressed
11	11	LOC_Os11g01439	14.88	262,011–256,827	139 bp	2793	931	Chloroplast unusual positioning protein, putative, expressed
12	11	LOC_Os11g36340	18.99	21,413,596–21,417,739	1.8 kb	1293	431	Lymphoid organ expressed yellow head virus receptor protein, putative, expressed
13	11	LOC_Os11g36740	30.82	21,687,447–21,691,214	1.8 kb	1305	435	DUF593 domain-containing protein, expressed

*Chr* chromosome, *Os Oryza sativa*, *CDS* coding sequence

In a similar way, LOC_Os01g73160, another gene, which is 1 kb away from the peak marker position of major QTL *qLTGS(I)*_*1–2*_ (16.57%) on chromosome 1, is the locus associated with ribosomal protein-small subunit (RPS-10), mainly involved in the abiotic stress tolerance mechanism and also in the development of the vegetative growth stage in leaves and shoots (Moin et al. 2016; Saha et al. 2017). To date, 34 RPS have been identified, which encode to 123 genes containing multiple copies of genes in rice. However, seven genes (RPS4, 7, 8, 9, 10, 19, and 26) were early responsive genes that were significantly upregulated under salinity stress (Kawasaki et al. 2001; Saha et al. 2017). Another major locus on chromosome 7, 4.0 kb away from the peak marker, encodes PHD-finger domain-containing protein locus (LOC_Os07g07690), encodes the transcription factor involved in submergence stress tolerance mechanism (Sharma et al. 2018).

### Putative candidate genes in co-localized M-QTLs

The co-localized QTLs are associated with multiple functional roles in the physiology related to ABA synthesis, responses to plant growth regulators, the formation of chloroplast, and tolerance of biotic and abiotic stresses. The major role of possible candidate genes on chromosome 1 (*LOC_Os01g37100*) encoding the RWP-RK domain is involved in early regulation of cellular responses to N supply and low phosphate availability (Castaings et al. 2009; Chardin et al. 2014). Chromosome 2 (3.83 Mb) is associated with *OsMADS29* - MADS-box family gene (*LOC_Os02g07430*), which encodes for a putative B-sister-type MIKCc transcription factor, mainly involved in the seed development. The *LOC_Os02g07430* is responsible for the regulation of starch biosynthesis during the process of seed development by regulating the cytokinin levels (Agarwal et al. 2016: Chen et al. 2013). Sharma et al. (2012) and Nayar et al. (2013) reported that the *OsMADS29* gene regulates the hormone homeostasis and starch filling in endosperm cells. Similarly, at 3.54 Mb, *LOC_Os03g06950* is responsible for auxin signaling pathways in shoot development and also early development of rice stamens (Yang et al. 2007; Wang et al. 2018). Another locus encodes to *LOC_Os03g29810*, which controls several functions in the synthesis of plastidic proteins in the chloroplast and regulates the photosynthesis mechanism (Dong et al. 2013; van Campen et al. 2016). Two genes on chromosome 6 (*LOC_Os06g17220* and *LOC_Os06g48300*) are responsible for sucrose synthetic activity and tolerance of anoxia condition during germination (Lasanthi-Kudahettige et al. 2007) and regulate the major ABA-dependent signaling component, which is involved in a high rate of germination (Bhatnagar et al. 2017). Singh et al. (2015) revealed that the *OsPP108* gene of the PP2C family significantly enhanced tolerance of drought, cold, and salinity during seed germination. On chromosome 8, *LOC_Os08g05440* is associated with resistance to biotic stresses and chloroplast degradation (van der Biezen and Jones 1998; Jiao et al. 2012). Lastly, *LOC_Os11g01439* on chromosome 11 is involved in the intracellular changes of chloroplast position (Oikawa et al. 2003) and has a potential role in the formation of protein bodies in the endosperm and the interaction of myosin (Peremyslov et al. 2013). Therefore, a further expression and fine mapping of potential candidate genes/alleles may provide valuable information for increasing LTS tolerance during the embryo burst stage and seedling growth stage in rice.

## Conclusions

Significant M-QTLs for different stages of germination and seedling growth-related traits under LTS conditions were identified in the specific genomic regions of different chromosomes. Exposure to cold stress for 16 days resulted in the identification of 24 ILs as the most promising lines with more than 90% germination. During the early growth stages of cold stress, GR, RL, SL, BM, and SVI were highly significant and strongly associated with each other. Of the 82 QTLs, five major M-QTLs were located on chromosomes 1, 5, and 7, and 16 genomic regions co-localized with two to four traits were also observed in the present study. The highest number of QTLs was located on chromosome 11, followed by chromosomes 1 and 6. Three possible candidate genes for major M-QTLs and 13 genes from co-localized M-QTL positions are associated with the combination of multiple traits. This genomic information is highly valuable for the fine mapping and validation of gene expression under LTS. However, promising ILs in the present study could be crossed with cold-sensitive elite cultivars to develop desirable recombinants for cultivation in temperate and high-altitude and high-latitude regions. In addition, designed QTL pyramiding will be carried out by inter-crossing the introgression lines and pooling the M-QTLs into pyramiding lines. For the use of MAS, there is an urgent need to fine-map the genomic region, identify genes, and alleles, and further develop functional markers for the improvement of cold tolerance in rice.

## Electronic supplementary material

ESM 1 (DOCX 18 kb)

## Abbreviations

ILs: Introgression lines
SNP: Single nucleotide polymorphism.
LTS: Low-temperature stress.
BC: Backcross breeding.
QTL: Quantitative trait loci
MAS: Marker-assisted selection
