# Supplementary material for: Identification of main-effect quantitative trait loci (QTLs) for low-temperature stress tolerance germination- and early seedling vigor-related traits in rice (Oryza sativa L.)
Source: Mol Breed. 2020 Jan 4;40(1):10. doi: 10.1007/s11032-019-1090-4 (PMC6944268; doi:10.1007/s11032-019-1090-4)
Supplement: Supplementary file 1 — (DOCX 18 kb) [file 11032_2019_1090_MOESM1_ESM.docx]

**Supplemental Table 1** Analysis of variance for all traits concerning conditions and stages

| **S. no.** | **Traits** | **MS genotypes**  **(df = 230)** | **MS error**  **(df = 230)** | **Pr(F)** | **CV (%)** |
| --- | --- | --- | --- | --- | --- |
| 1 | LTG-I Control | 1091.37 | 6.23 | <0.01 | 5.34 |
| 2 | LTG-II Control | 871.96 | 6.58 | <0.01 | 3.32 |
| 3 | LTG-III Control | 871.96 | 6.58 | <0.01 | 3.32 |
| 4 | RGP (I-II) Control | 673.15 | 9.47 | <0.01 | 10.05 |
| 5 | RGP (II-III) Control | 245.49 | 0.78 | <0.01 | 12.97 |
| 6 | LTG-I Stress | 245.49 | 0.87 | <0.01 | 12.8 |
| 7 | LTG-II Stress | 1127.9 | 3.9 | <0.01 | 4.44 |
| 8 | LTG-III Stress | 1547.97 | 8.71 | <0.01 | 4.86 |
| 9 | RGP (I-II) Stress | 789.03 | 4.38 | <0.01 | 5.61 |
| 10 | RGP (II-III) Stress | 466.05 | 8.86 | <0.01 | 18.38 |
| 11 | AGP-I Control | 874.7 | 7.43 | <0.01 | 19.61 |
| 12 | AGP-II Control | 1396.7 | 26.8 | <0.01 | 8.95 |
| 13 | AGP-III Control | 1536.8 | 29.8 | <0.01 | 7.38 |
| 14 | LTGS-I Control | 0.08 | 0.001 | <0.01 | -3.27 |
| 15 | LTGS-II Control | 0.18 | 0.001 | <0.01 | -10.78 |
| 16 | LTGS-III Control | 0.68 | 0.003 | <0.01 | -14.48 |
| 17 | BW Control | 0.16 | 0.002 | <0.01 | 7.75 |
| 18 | BW Stress | 0.3 | 0 | <0.01 | 6.99 |
| 19 | BMSI | 0.21 | 0.006 | <0.01 | -11.44 |
| 20 | RL-I Control | 473.3 | 4.25 | <0.01 | 4.96 |
| 21 | RL-II Control | 584.15 | 3.54 | <0.01 | 3.25 |
| 22 | RL-III Control | 2434.66 | 1.017 | <0.01 | 3.21 |
| 23 | RGI (I-II) Control | 375.28 | 5 | <0.01 | 13.77 |
| 24 | RGI (II-III) Control | 2277.6 | 2.96 | <0.01 | -6.5 |
| 25 | RL-I Stress | 673.15 | 9.47 | <0.01 | 10.05 |
| 26 | RL-II Stress | 198.07 | 0.92 | <0.01 | 7.75 |
| 27 | RL-III Stress | 366.8 | 1.29 | <0.01 | 6.92 |
| 28 | RGI (I-II) Stress | 63.81 | 1.04 | <0.01 | 18.71 |
| 29 | RGI (II-III) Stress | 74.16 | 1.69 | <0.01 | 22.33 |
| 30 | RLSI-I | 0.1 | 0 | <0.01 | -2.27 |
| 31 | RLSI-II | 0.1 | 0.06 | <0.01 | -2.17 |
| 32 | RLSI-III | 0.31 | 0.001 | <0.01 | -3.94 |
| 33 | SL-I Control | 124.6 | 2.23 | <0.01 | 5.45 |
| 34 | SL-II Control | 316.6 | 4.3 | <0.01 | 3.78 |
| 35 | SL-III Control | 2181.7 | 1.97 | <0.01 | 4.62 |
| 36 | SGI (I-II) Control | 266.45 | 5.35 | <0.01 | -8.42 |
| 37 | SGI (II-III) Control | 2108.9 | 4.18 | <0.01 | -24.47 |
| 38 | SL-I Stress | 49.74 | 0.1 | <0.01 | 5.7 |
| 39 | SL-II Stress | 204.25 | 0.86 | <0.01 | 7.29 |
| 40 | SL-III Stress | 438.55 | 1.42 | <0.01 | 6.66 |
| 41 | SGI (I-II) Stress | 39.37 | 0.95 | <0.01 | 13.68 |
| 42 | SGI (II-III) Stress | 99.45 | 1.97 | <0.01 | 24.45 |
| 43 | SLSI-I Control | 0.1 | 0.0001 | <0.01 | -2.49 |
| 44 | SLSI-II Control | 0.107 | 0 | <0.01 | -2.64 |
| 45 | SLSI-III Control | 0.3 | 0 | <0.01 | -5.08 |
| 46 | SVI-I Control | 888.01 | 6.3 | <0.01 | 7.38 |
| 47 | SVI-II Control | 1856.8 | 17.86 | <0.01 | 4.78 |
| 48 | SVI-III Control | 6262.5 | 8.38 | <0.01 | 5.86 |
| 49 | SVI-I Stress | 12.2 | 0.05 | <0.01 | 18.05 |
| 50 | SVI-II Stress | 313.4 | 1.25 | <0.01 | 8.43 |
| 51 | SVI-III Stress | 1046.6 | 4.16 | <0.01 | 8.05 |

**MS =** Mean squares/variances; **Pr** = Probability; **CV** = Coefficient of variation
